# MLPA-based genotype–phenotype analysis in 1053 Chinese patients with DMD/BMD

**DOI:** 10.1186/1471-2350-14-29

**Published:** 2013-03-01

**Authors:** Juan Yang, Shao Y Li, Ya Q Li, Ji Q Cao, Shan W Feng, Yan Y Wang, Yi X Zhan, Chang S Yu, Fei Chen, Jing Li, Xiao F Sun, Cheng Zhang

**Affiliations:** 1 Department of Neurology, The First Affiliated Hospital, Sun Yat-sen University, 58 Zhong Shaner Road, Guangzhou City, 510080, People’s Republic of China; 2 Department of Reproductive Medicine, The Third Affiliated Hospital of Guangzhou Medical College, Guangzhou City, 510150, People’s Republic of China; 3 Institute of Population Research, Peking University, No.5 Yiheyuan Rd., Haidian District, Beijing City, 100080, People’s Republic of China; 4 Guangzhou Kingmed Diagnostics Center, Guangzhou City, 510330, People’s Republic of China

**Keywords:** Duchenne muscular dystrophy, Becker muscular dystrophy, *DMD* gene, MLPA

## Abstract

**Background:**

Large-scale analysis of the transmission, mutation characteristics and the relationship between the reading frame and phenotype of the *DMD* gene has previously been performed in several countries, however, analogous studies have yet to be performed in Chinese populations.

**Methods:**

Clinical data from 1053 Chinese patients with DMD/BMD were collected, and the *DMD* gene was tested by MLPA in all patients and 400 proband mothers. In 20 patients with negative MLPA, sequencing was also performed.

**Results:**

We found that 27.50% of cases had a family medical history of DMD/BMD, and large rearrangements were identified in 70.56% of the probands, of which 59.35% and 11.21% were deletions or duplications, respectively. The carrier status of the mothers in the study was determined to be 50.75%, and it was established that the *DMD* mutation was inherited from the mother in 51.72% of the probands. Exons 45–54 and 3–22 were the most frequently deleted regions, and exons 3–11 and 21–37 were the most prevalently duplicated regions of the gene. Breakpoints mainly occurred in introns 43–55 for deletion mutations and in introns 2 and 7 for duplication mutations. No breakpoints were found at the 5^′^ or 3^′^ end of introns 31, 35, 36, 40, 65, 68, and 74–78 in any of the deletion or duplication mutations. The reading frame rule held true for 86.4% of the DMD patients and 74.55% of the BMD patients.

**Conclusion:**

It is essential to increase physicians’ understanding of DMD/BMD, to promote scientific information, and to increase awareness in regards to genetic counseling and prenatal diagnosis in pedigrees with a family history of the disease, particularly in families with small *DMD* lesions in China. In addition, such a large-scale analysis will prove to be instructive for leading translational studies between basic science and clinical medicine.

## Background

Duchenne muscular dystrophy (DMD) and its less severe allelic form, Becker muscular dystrophy (BMD), are common X-linked recessive neuromuscular diseases caused by mutations in the *DMD* gene. This gene consists of 79 exons and encodes a 14.6 kb mRNA, which is mainly expressed in skeletal muscle and myocardial and brain tissue [1-3]. The estimated incidence of DMD and BMD is 1/3500 and 1/18,000 of male live births, respectively [4,5]. Levels of dystrophin protein are remarkably reduced or absent in DMD (<3% of normal), whereas BMD patients have 10–40% of the normal amount of dystrophin but of an abnormal molecular weight [6,7]. DMD/BMD patients first present with either complaints of progressive proximal muscular weakness and atrophy of the limbs or remarkably elevated transaminase levels upon examination. Because many clinicians lack a complete understanding of the disease, there are often several patients with DMD/BMD within a family pedigree due to a late diagnosis of the disease. To date, no effective therapy is available for DMD/BMD patients. Therefore, it is essential to perform genetic counseling and prenatal screening to prevent passing on the disease. Furthermore, it will be instructive to lead translational studies between basic science and clinical medicine by large-scale analyses of *DMD* gene defects. Recently, large-scale analyses of the transmission, the mutation characteristics and the relationship between the reading frame and phenotype of the *DMD* gene have been performed in several countries. However, analogous studies have yet to be performed in Chinese populations.

In this study, multiplex ligation-dependent probe amplification (MLPA)-based genotype–phenotype analysis was performed in 1053 Chinese patients with DMD/BMD.

## Methods

### Subjects

A total of 1053 male patients in China were studied, including 951 with DMD, 96 with BMD and 6 with IMD. Of the 1053 cases, 793 had detailed information about their family history, and in which 218 had a family history. In addition to these cases, 400 mothers of probands were included in this study. Diagnosis was based on clinical presentations, markedly elevated serum creatine kinase levels, electromyography and muscle biopsy. This study was approved by the institutional review board of Sun Yat-sen University Affiliated First Hospital, and informed consent was obtained from all participants.

### Methods

### MLPA

Genomic DNA was extracted from peripheral blood according to standard procedures (QIAamp DNA Blood Mini Kit Handbook,QIAGEN), and the *DMD* gene was detected by MLPA [8] according to instructions of SALSA MLPA probemix P034-A3/P035-A3 DMD/Becker (MRC Holland) for use in all patients and proband mothers . All initial data of MLPA were analyzed by Excel-based Coffalyser, the allele copy numbers were determined by cut-off values. The MLPA results from male patients were initially assessed visually for the detection of deletions, as the absence of *DMD* specific peaks. Absence of *DMD* peaks corresponding to two or more contiguous exons was taken to represent a genuine deletion and no further investigations were performed. The absence of only one *DMD* peak in males, corresponding to a single exon, was investigated further using PCR primers flanking the exon in question designed using Primer Premier 5.0. If a deletion could not be confirmed by PCR, the potential PCR products of these exons were sequenced. In females, the absence of a single peak was investigated by direct sequencing. MLPA was replicated to confirm them in any case ambiguous duplications or amplifications were found.

### Sequencing

PCR amplification and direct sequencing were performed using forward and reverse primers [9] complementary to all 79 exons and exon-intron junctions (ABI Prism 3100 Genetic Analyzer, Applied Biosystem, Foster City, USA). Mutation nomenclature of small lesions was performed according to standard conventions [10].

## Result

### Detection of *DMD* gene mutations with MLPA

**Figure 1:**
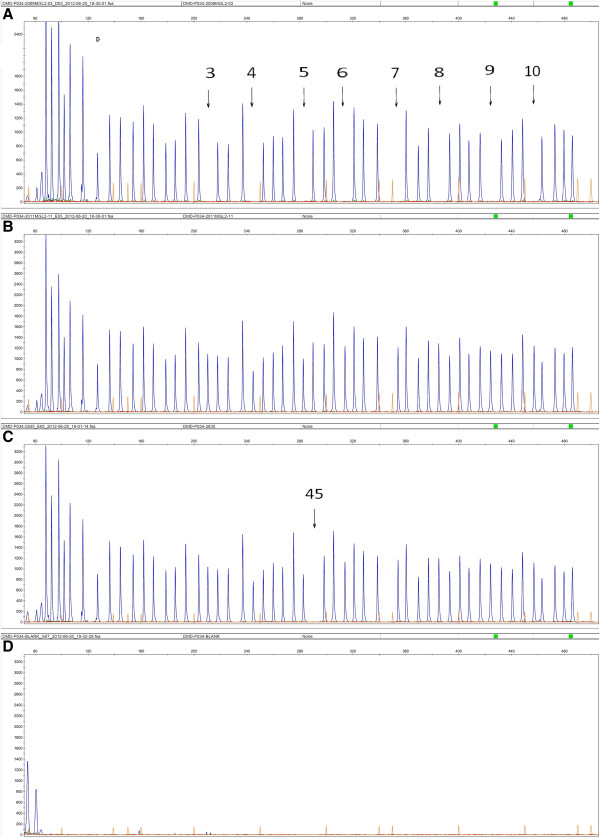
**Deletion of exon 45 of the *DMD *gene in one affected boy (C).** (**A**) Positive control (Deletion of exons 3–10), (**B**) Normal control and (**D**) blank control.

Large rearrangements were identified in 743 of the 1053 probands (743/1053, 70.56%) using MLPA. The rearrangements consisted of 625 large deletions and 118 large duplications spanning one or more exons, representing 59.35% (625/1053) and 11.21% (118/1053) of all mutations identified in this study, respectively. Among 1053 probands, no *DMD* gene defects were identified by MLPA in 310 cases. There were 576 DMD (576/625, 92.16%), 46 BMD (46/625, 7.36%) and 3 IMD (3/625, 0.48%) probands with deletions, and 106 DMD (106/118, 89.83%), 9 BMD (9/118, 7.63%) and 3 IMD (3/118, 2.54%) probands with duplications. Figure 1 shows an example of MLPA-detected single-exon deletions. 

### Small fragment deletions are predominant in the *DMD* gene

Among the 1053 probands, single-exon deletions (150) were the most frequent, followed by deletions of 3 exons, deletions of 2 exons and deletions of 5 exons. Our results showed that deletions of 6 exons or fewer accounted for 45.68% (481/1053) of cases. Deletions of 10 exons or fewer accounted for 53.18% (560/1053) of the probands.

### Exons 45–54 and exons 3–22 are the most commonly deleted regions

Single-exon deletions represented up to 24.00%(150/625)of the 625 deletions identified and mainly occurred in exons 44–45 and 50–54. Exons 51 and 45 were the most commonly deleted exons in single-exon deletions, accounting for 5.44% (34/625) and 4.64% (29/625) of deletions, respectively. Of 475 cases with multi-exon deletions, a 6-exon deletion of 45–50 was the most prevalent (36), followed by a 3-exon deletion of 48–50 (30), 2-exon deletion of 49–50 (24), and 3-exon deletion of 45–47 (23). However, there were 21 cases with 8-exon deletions of 45–52 and 20 cases with 2-exon deletions of 46–47, respectively. Exons 45–54 and exons 3–22 were the most commonly deleted regions. Of all the exons, exon 50 was the most frequently deleted, followed by exons 49, 48, 47, and 46. We also observed 1 case that resulted from all 79 exons.

### Small fragment duplications are prevalent in the *DMD* gene

**Figure 2:**
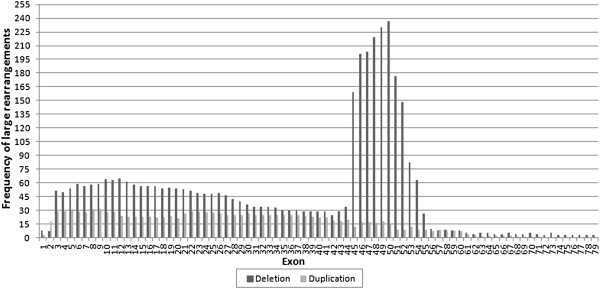
**Arrangement frequency of each exon in the *DMD *gene (625 deletions and 118 duplications).**

Single-exon duplications accounted for 20.34% (24/118) of the 118 duplications, of which the duplication of exon 2 (12) was the most prevalent, followed by the 5-exon duplication of 3–7 (7). Our results showed that a duplication of 6 exons or fewer accounted for 51.69% of all duplications (61/118). All duplications involving 10 exons or fewer accounted for 67.80% (80/118) of the duplications identified in the probands. Hotspot regions of duplication were located in exons 3–11 and 21–37. Exons 8 and 9 were the most frequently duplicated exons, whereas no duplications were found in each exon from 72 to 79. Furthermore, 7 cases of two-tandem-duplication and 1 case of three-tandem-duplication were found in our study. Figure 2 showed rearrangement frequency of each exon in the *DMD* gene. 

### Breakpoint distribution

Deletion breakpoints were clustered at introns 43–55 of the *DMD* gene. Intron 50 was by far the most frequently involved (14.40%, 180/1250), preferentially as a starting breakpoint (66.67%, 120/180), followed by introns 44 (12.88%, 161/1250), 45 (8.48%, 106/1250), 47 (8.32%, 104/1250) and 52 (7.20%,90/1250). No deletion breakpoints were observed in introns 15, 24, 31, 35, 36, 38–40, 59, 65, 68 and 74–78. As an ending breakpoint, intron 44 was the most frequently involved (11.44%, 143/1250). Intron 45 was the second most frequently observed 3’ breakpoint site (5.92%, 74/1250), both introns 47 and 50 was the third (60/1250, 4.80%), followed by introns 2 (3.60%, 45/1250) and 48 (3.12%, 39/1250). The proportion of starting breakpoints was reduced successively in introns 50 (9.60%, 120/1250), 52 (6.24%, 78/1250), 51 (3.60%, 45/1250), 47 (3.52%, 44/1250), 54 (3.12%, 39/1250) and 45 (2.56%, 32/1250).

**Figure 3:**
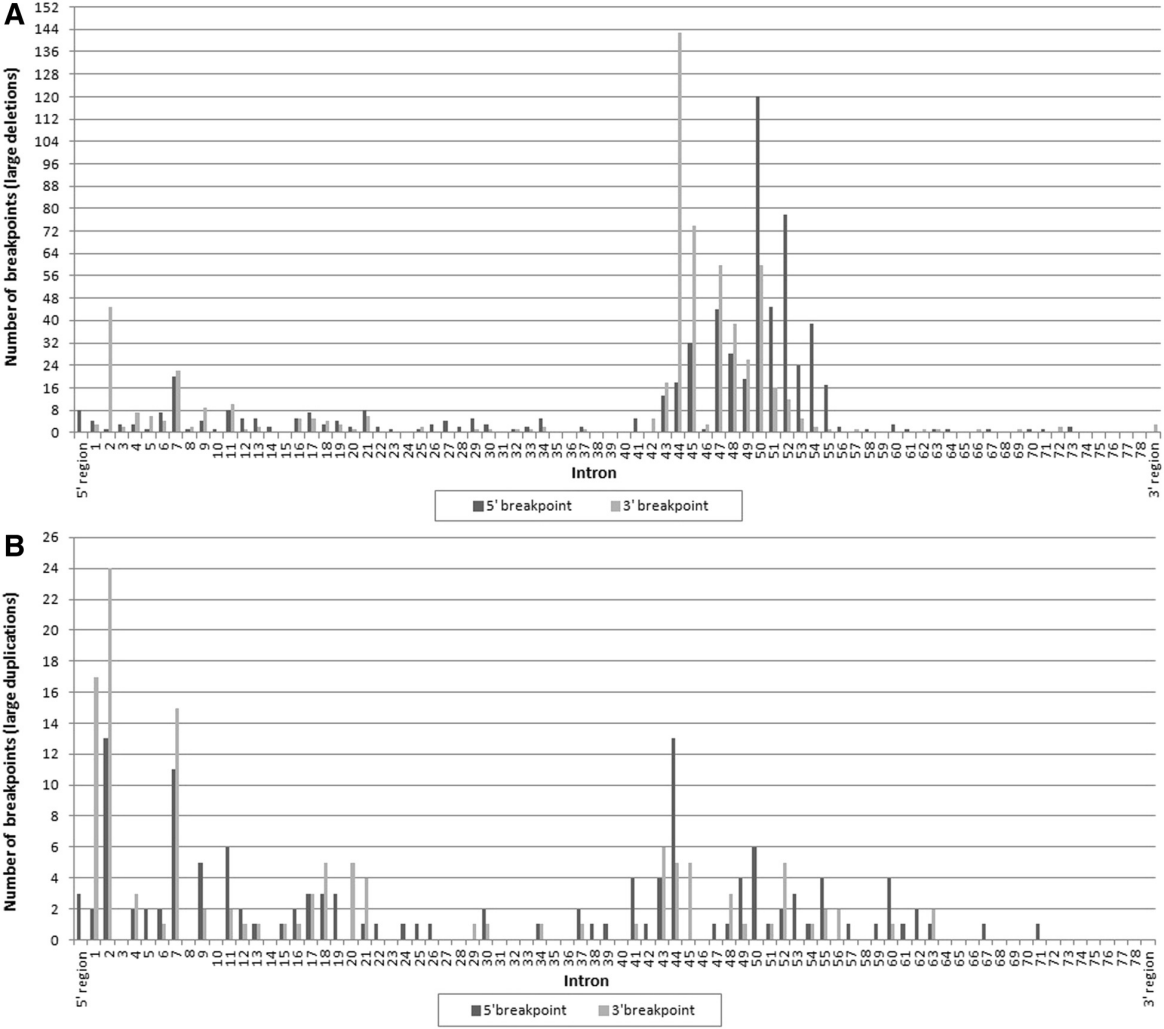
**Distribution of intronic breakpoints of large rearrangements within the *DMD *gene.** (**A**) Intronic deletions breakpoints and (**B**) Intronic duplications breakpoints. The number of starting breakpoint (5^′^ breakpoint) and ending breakpoint (3^′^ breakpoint) of either deletion or duplication localized in each intron is indicated.

Duplication breakpoints were clustered at the 5^′^ end of the *DMD* gene. Intron 2 was the most frequently involved (14.57%, 37/254), followed by introns 7 (10.24%, 26/254), 1 (7.48%, 19/254) and 44 (7.09%, 18/254). No duplication breakpoints were found in introns 3, 8, 10, 14, 23, 27, 28, 31–33, 35, 36, 40, 46, 58, 64–66, 68–70 and 72–78. Intron 2 was most frequently involved in both starting and ending breakpoints and accounted for 5.12% (13/254) and 9.45% (24/254) of each, respectively. Corresponding to the duplication frequency, a high proportion of ending breakpoints were present in introns 1 (6.69%, 17/254), 7 (5.91%, 15/254) and 43 (2.36%, 6/254), whereas intron 44 contained a high proportion of starting breakpoints (5.12%, 13/254). Figure 3 showed distribution of intronic breakpoints of large rearrangements within the *DMD* gene. 

### Sequencing of probands with negative MLPA

Considering the high cost related to the large *DMD* gene, in this study, only 20 unrelated probands with negative MLPA were selected randomly for further investigation. Small lesions were identified by sequencing for these probands, of which 8 cases were nonsense point mutations, 7 cases were splice-site deletion/substitution mutations and 5 cases were missense mutations. Sequencing results were listed in Table 1. 

**Table 1:** Sequencing results of 20 male probands with negative MLPA

**No.**	**Phenotype**	**Family history**	**Exon/Intron**	**Sequencing results**	**Change of protein**	**Carrier status of mother**
1	DMD	Unknown	Exo 2	c.70 T > C	p.Trp24Arg	Unknown
2	DMD	Unknown	Exo 2	c.77A > G	p.Asn26Ser	Yes
3	DMD	No	Exo 6	c.436C > T	p.Gln146X	No
4	DMD	Unknown	Int 16	c.1993-37 T > G/	Unknown	Unknown
			Int 49	c.7200 + 53C > G		
5	DMD	Unknown	Exo 19	c.2302C > T	p.Arg768X	Yes
6	DMD	Unknown	Int 20	c.2623-34C > T/	Unknown	Yes
			Int 66	c.9649 + 15 T > C		
7	DMD	Unknown	Int 21	c.2804-1 G > T	Unknown	No
8	DMD	Unknown	Exo 29	c.4057 G > T	p.Glu1353X	Unknown
9	DMD	Unknown	Exo 32	c.4375C > T	p.Arg1459X	Yes
10	DMD	Yes	Int 32	c.4518 + 5 G > A	Unknown	Yes
11	DMD	Unknown	Exo 34	c.4729C > T	p.Arg1577X	Unknown
12	DMD	Unknown	Exo 37	c.5234 G > A	p.Arg1745His	Unknown
13	DMD	Unknown	Exo 44	c.6373C > T	p.Gln2125X	Yes
14	DMD	Unknown	Exo 48	c.7096C > A	p.Gln2366Lys	Unknown
15	DMD	No	Exo 52	c.7657C > T	p.Arg2553X	Yes
16	DMD	Yes	Int 61	c.9164-3_9164-1delCAG	Unknown	Yes
17	DMD	Unknown	Int 66	c.9649 + 15 T > C	Unknown	Yes
18	DMD	Unknown	Exo 68	c.9934A > C	p.Lys3312Thr	Yes
19	DMD	Unknown	Exo 70	c.10141C > T	p.Arg3381X	Unknown
20	DMD	Unknown	Int 70	c.10223 + 1 G > A	Unknown	Yes

### Transmission

Among a total of 387 DNA samples from the mothers of 379 unrelated cases (311 with deletions, 68 with duplications), 12 cases with deletions in 6 families (2 cases per family), and 4 cases with duplications in 2 families (2 cases per family), 192 carriers were detected by MLPA: 137 with deletions, 55 with duplications. In addition, among 13 DNA samples from the mothers of 13 unrelated cases with small lesions, 11 mothers were identified to be carriers by sequencing. Combining all mutations, 50.75% [(192 + 11)/(387 + 13)] of the mothers were carriers, and it was established that the *DMD* gene mutation was inherited from the mother in 51.72% [(192 + 6 + 2 + 11)/(379 + 12 + 4 + 13)] of cases. Therefore, cases resulting from de novo mutations represented up to 48.28% (1–51.72%).

### Reading frame and phenotype

Excluding 10 DMD patients with deletions in exon 1 or exon 79, of the 625 deletions, 566 resulted in DMD, including 70 cases of in-frame and 496 cases of frame-shift mutations. The 46 BMD patients included 36 cases of in-frame and 10 cases of frame-shift mutations, and the 3 IMD cases resulted from frame-shift mutations.

Excluding 3 DMD patients with duplications in exon 1 or exon 79, of the 118 duplications, 103 resulted in DMD, including 21 cases of in-frame and 82 cases of frame-shift mutations. The 9 BMD cases were caused by either in-frame (5) or frame-shift (4) mutations, and the 3 IMD cases resulted from frame-shift mutations. Deletions contradicting the reading frame rule were clustered at the 5′ end of the *DMD* gene, whereas duplications were clustered at the 3′ end of the gene (See Table 2). 

**Table 2:** Large rearrangements contrary to reading frame rule

**Genotype**	**IF/OF**	**Phenotype (No.)**
DelEx3-6	OF	DMD(1)/BMD(2)
DelEx3-7	OF	DMD(15)/BMD(1)
DelEx3-10	IF	DMD (1)
DelEx3-13	IF	DMD (1)
DelEx3-26	IF	DMD (2)
DelEx3-27	IF	DMD (2)
DelEx3-29	IF	DMD (1)/BMD (1)
DelEx3-30	IF	DMD (1)
DelEx3-33	IF	DMD (1)
DelEx3-37	IF	DMD (1)
DelEx3-44	IF	DMD (3)
DelEx5-33	IF	DMD (1)
DelEx6-48	IF	DMD (1)
DelEx8-21	IF	DMD(3)/BMD(1)
DelEx10-30	IF	DMD (1)
DelEx10-34	IF	DMD (1)
DelEx10-41	IF	DMD (1)
DelEx10-44	IF	DMD (1)
DelEx12-43	IF	DMD (1)
DelEx13-34	IF	DMD (1)
DelEx14	IF	DMD (1)
DelEx17-30	IF	DMD (1)
DelEx26-34	IF	DMD (1)
DelEx34	IF	DMD (1)
DelEx44	OF	DMD(9)/BMD(1)/IMD(1)
DelEx44-45	IF	DMD (1)
DelEx45-46	IF	DMD (1)
DelEx45-47	IF	DMD(12)/BMD(11)
DelEx45-48	IF	DMD(9)/BMD(2)
DelEx45-49	IF	DMD(4)/BMD(2)
DelEx45-52	OF	DMD(19)/BMD(1)/IMD(1)
DelEx45-53	IF	DMD(3)/BMD(1)
DelEx45-55	IF	DMD (1)
DelEx48	IF	DMD (3)
DelEx48-49	IF	DMD (2)/BMD(1)
DelEx48-50	OF	DMD (29)/BMD(1)
DelEx48-51	IF	DMD (1)/BMD(1)
DelEx48-67	IF	DMD (1)
DelEx50	OF	DMD (15)/BMD(1)
DelEx51	OF	DMD (33)/BMD(1)
DelEx51-52	IF	DMD (3)/BMD(2)
DelEx51-53	OF	DMD (8)/IMD(1)
DelEx51-55	OF	DMD (4)/BMD(1)
DelEx63	OF	BMD(1)
DelEx64	IF	DMD (1)
DupEx2	OF	DMD (11)/BMD(1)
DupEx2-7	IF	DMD (2)
DupEx2-44	OF	IMD (1)
DupEx3-4	IF	DMD (2)
DupEx3-5	IF	DMD (1)
DupEx3-6	OF	DMD (1)/BMD(1)
DupEx3-7	OF	DMD (5)/BMD(1)/IMD (1)
DupEx3-9&17-41	IF	DMD (1)
DupEx3-15	IF	DMD (1)
DupEx3-18	IF	DMD (1)
DupEx3-39	IF	DMD (1)
DupEx3-41	IF	DMD (1)
DupEx3-44	IF	DMD (1)
DupEx8-44	OF	BMD (1)
DupEx19-43&49-51	OF	IMD (1)
DupEx19-44	IF	DMD (2)
DupEx21-25	IF	DMD (1)
DupEx21-37	IF	DMD (2)
DupEx21-44	IF	DMD (1)
DupEx31-44	IF	DMD (1)
DupEx45-49	IF	DMD (1)
DupEx49-55	IF	DMD (1)
DupEx53-63	IF	DMD (1)

## Discussion

Though no other methods were used to detect large rearrangement of the *DMD* gene in our study, several studies have shown that MLPA is useful to quantitatively detect mutations in the *DMD* gene, not only for identifying deletions but also for duplications and female carriers [11,12]. More mutations can be detected when MLPA is integrated with sequencing. Our results indicate that 59.35% of the probands carry deletions and 11.21% carry duplications, which resulted in a sensitivity of detecting DNA rearrangement by MLPA of 70.56%. In agreement with similar studies, DMD is the most common phenotype resulting from large rearrangements or small lesions of the *DMD* gene. Most large rearrangements result in deletion mutations, whereas small lesions are often nonsense mutations [13]. As an economical, rapid, sensitive and easy genetic testing method, MLPA should be considered as the initial test for suspected DMD/BMD patients; it should also be considered to provide better genetic counseling to women with a family history of DMD/BMD. Certainly, sequencing can be further used to test for *DMD* gene defects in instances where MLPA did not detect a mutation.

In China, DMD/BMD patients present initially at the hospital with either progressive proximal muscular weakness and atrophy of the limbs or significantly elevated transaminase level on examination. Because many physicians lack a complete understanding of the disease, there are often several patients with DMD/BMD within the same family pedigree due to a late diagnosis of the disease. In our study, 27.49% of the probands had a family medical history of the disease, and 50.75% of the mothers were carriers (small lesion>duplication>deletion). It was also established that the *DMD* gene mutation was inherited from the mother in 51.72% of cases, whereas 48.28% of the mutations were de novo. These data demonstrate it is essential to increase physicians’ understanding of DMD/BMD, to promote scientific information, and to increase awareness in regards to genetic counseling and prenatal diagnosis in pedigrees with a family history of the disease, particularly in families with small *DMD* lesions. In addition, it will be important to prioritize DMD/BMD as a prenatal screening project in the future.

Exons 2–20 and 44–53 had been previously reported as hotspot regions in the *DMD* gene. In this study, we analyzed the distribution of individual exons based on their frequency of deletion or duplication. Our data indicate that the deletion regions occurring in exons 45–54 and exons 3–22 are the most frequent, that deletions in exons 1–2 and 56–79 were extraordinarily rare. In agreement with previous work, small regional deletions were found to be more common in the *DMD* gene, and exon 51 was the most prevalent in single-exon deletions. Previous studies have suggested that there is a relationship between repetitive sequences and breakpoints within introns: the more the repetitive sequence, the more breakpoints there are and the higher the incidence of mutation. Intron 44 is the most frequent starting breakpoint, followed by intron 47 and intron 50. The proportion of ending breakpoints compared to starting breakpoints within introns gradually increases from intron 47 to intron 52. There is a relationship between the clustering breakpoints and the formation of deletion hotspots [13]. In contrast to the literature, our study found deletion breakpoints mainly clustered at the introns 43–55 of the *DMD* gene and duplications at the 5^′^ end of the *DMD* gene. Deletion breakpoints mainly occurred at the 5^′^ terminal of introns 43–45, 47–55 and 7 and at the 3^′^ terminal of introns 43–45, 47–51, 2 and 7. Intron 44 is the most frequent ending breakpoint, followed by introns 45, 50/47, 2, 48 and 49. The frequency of breakpoints reduced with each successive exon, and no increasing trend was found from intron 47 to intron 52. A similar pattern was not found in duplication breakpoints. Whether it was deletion or duplication, no starting breakpoints or ending breakpoints were found in introns 31, 35, 36, 40, 65, 68and 74–78. We speculate that the distribution of breakpoints is one of the determining factors leading to the formation of hotspot mutation regions. In this study, the distribution of duplications is in agreement with other countries. It was reported that the predominant cause of duplications in the *DMD* gene is the unequal crossing over between sister chromatids [14]. It is possible that interchromosomal events may be another mechanism of genomic duplication because no nucleotide differences were found in the duplicated alleles [15]. Recently, the occurrence of tandem duplication via non-homologous end joining has been proposed as a mechanism for exon 2 duplications [16].

In general, the severity of the phenotype depends on the occurrence of a translation reading frame disruption and the premature termination of protein synthesis. The reading frame rule has been shown to hold true for 96% and 93% of the mutations in DMD and BMD patients, respectively [13]. In our study, there were 84.75% [(496 + 82)/(10 + 566 + 3 + 103)] of DMD and 74.55% [(36 + 5)/(46 + 9)] of BMD cases, which were consistent with the reading frame rule. The deletion beginning with exon 3 represented 35.56% of the exceptions in DMD patients. Several groups have reported that 41% of cases with deletions in exons 3–7 presented with BMD due to the correction of alternative splicing events, usage of the potential promoter within the intron, and recoding after ribosomal frameshift [17-19]. However, excluding 1 case of BMD, the other 15 cases with the deletion of exons 3–7 presented with DMD in our study. Tuffery-Giraud et al. reported a case with a deletion of exon 48 that did not present with any neuromuscular complaints or neuromuscular physical signs by 9 years of age [13]. However, 3 cases in our study with a single exon deletion of exon 48 presented with DMD. In addition to differences between unrelated patients, variable clinical phenotypes were observed in cases with identical gene mutations, even within the same pedigree. The deletion of exons 13–29 was found in a large family in which two male patients were mildly affected with BMD, whereas three other family members aged 2, 12, and 39 were clinically unaffected [13]. Multiple mechanisms may have a role in modulating these differences, including recoding after ribosomal frameshift, unusual alternative splicing [20,21], usage of novo promoters, exon skipping subsequent to special exon mutation and somatic mosaicism [13]. One study found that each exon duplicated in the *DMD* gene was incorporated into the fully spliced mRNA and that duplicated fragments were present in tandem to their respective fragments [15]. However, it is challenging to predict clinical phenotypes at the genomic level using the reading frame rule for unknown effects of inserted fragments and several complex duplication rearrangements or translocations on the structure and function of dystrophin. Therefore, further studies are required to determine precise genotype/phenotype correlations, and reading frame theory should be further refined.

Exon skipping is another therapy for DMD that can transform DMD into BMD, which is based on the recovery of the reading frame induced by alternative splicing of antisense oligonucleotides. Exon skipping has been confirmed as an effective DMD treatment by animal experiments and clinical trials [22,23]. Presently, only patients with deletions of certain exons can benefit from the development of antisense oligonucleotides. The strategy of exon skipping needs to be further improved to account for variations in clinical phenotype and sensitivity to antisense oligonucleotides in different patients and to account for the specific effects of each integral exon on the structure and function of dystrophin. More patients may benefit from individual exon skipping therapies following a comprehensive understanding of the correlation between genotypes and phenotypes under the guidance of large-scale genetic epidemiological studies.

## Conclusion

Considering a large number of DMD/BMD cases have family medical history in China, it is essential to increase physicians’ understanding of DMD/BMD, to promote scientific information, and to increase awareness in regards to genetic counseling and prenatal diagnosis in pedigrees with a family history of the disease, particularly in families with small *DMD* lesions in China. In addition, such a large-scale analysis will prove to be instructive for leading translational studies between basic science and clinical medicine.

## Competing interests

The authors declare that they have no competing interests.

## Authors’ contributions

JY contributed to conception and design, analysis of data of *DMD* gene, and drafted the manuscript. YQL, JQC, SWF, YYW, FC, and JL are responsible for acquisition of clinical data. XFS, CSY, SYL, and YXZ performed the *DMD* gene study through MLPA or sequencing. CZ supervised all the work, conceived of the study, participated in its design and coordination, and helped to draft the manuscript. All authors read and approved the final manuscript.

## Authors’ information

Juan Yang and Shao Y Li are co-first author.

## Pre-publication history

The pre-publication history for this paper can be accessed here:

http://www.biomedcentral.com/1471-2350/14/29/prepub
